# Bicomponent Cellulose Fibrils and Minerals Afford Wicking Channels Stencil-Printed on Paper for Rapid and Reliable Fluidic Platforms

**DOI:** 10.1021/acsapm.1c00856

**Published:** 2021-10-05

**Authors:** Katariina Solin, Maryam Borghei, Monireh Imani, Tero Kämäräinen, Kaisa Kiri, Tapio Mäkelä, Alexey Khakalo, Hannes Orelma, Patrick A. C. Gane, Orlando J. Rojas

**Affiliations:** † Department of Bioproducts and Biosystems, School of Chemical Engineering, Aalto University, Vuorimiehentie 1, FI-00076 Espoo, Finland; ‡ VTT Technical Research Centre of Finland Ltd., Functional Cellulose, Tietotie 4E, FI-02044 Espoo, Finland; § VTT Technical Research Centre of Finland Ltd., Micronova, Tietotie 3, FI-02150 Espoo, Finland; ∥ The Bioproducts Institute, Departments of Chemical and Biological Engineering, Chemistry and Wood Science, University of British Columbia, 2360 East Mall, Vancouver, BC V6T 1Z4, Canada

**Keywords:** fluidic channel, stencil printing, liquid wicking materials, paper-based microfluidics, multisensing assay

## Abstract

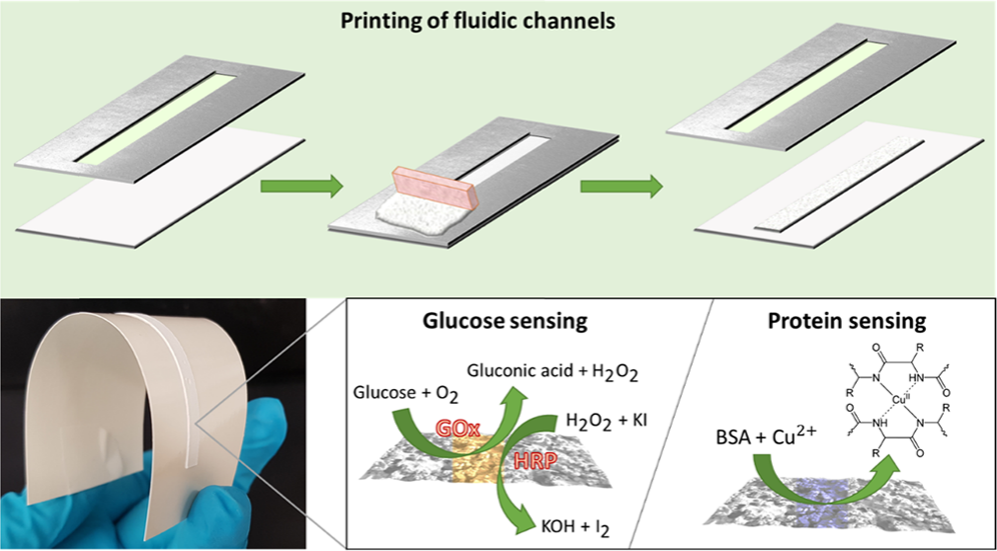

Flexible and easy-to-use microfluidic systems are suitable options for point-of-care diagnostics. Here, we investigate liquid transport in fluidic channels produced by stencil printing on flexible substrates as a reproducible and scalable option for diagnostics and paper-based sensing. Optimal printability and flow profiles were obtained by combining minerals with cellulose fibrils of two different characteristic dimensions, in the nano- and microscales, forming channels with ideal wettability. Biomolecular ligands were easily added by inkjet printing on the channels, which were tested for the simultaneous detection of glucose and proteins. Accurate determination of clinically relevant concentrations was possible from linear calibration, confirming the potential of the introduced paper-based diagnostics. The results indicate the promise of simple but reliable fluidic channels for drug and chemical analyses, chromatographic separation, and quality control.

## Introduction

Inexpensive and portable microfluidic technologies that require minimum sample preparation are highly desirable for point-of-care (POC) diagnostics, environmental and food quality control, and lab-on-chip analytical devices.^1,2^ Given their low cost, lightweight, and accessibility, paper-based microfluidic systems have been proposed.^3−6^ The latter has been used in litmus testing, chromatography, and lateral flow devices such as those used for pregnancy tests.^7,8^

Microfluidic devices are commonly based on nitrocellulose membranes. The popularity of nitrocellulose is primarily due to its ability to bind proteins irreversibly; in addition, it enables a good signal-to-noise ratio.^7^ However, the drawbacks of nitrocellulose include its high flammability, susceptibility to humidity, short shelf life, and low strength.^7,9^ Due to their hydrophobicity, commercial nitrocellulose flow membranes often require surfactants, which might cause reagent incompatibility and limit protein binding.^7^ Furthermore, the use of nitrocellulose or paper in lateral flow assays may involve a setup that requires adhesives; depending on the type, they may block the pores of the substrate and prevent application in printable electronics.

Alternatively, cellulose filters and chromatography paper are also used, following cutting, physical, or chemical patterning; these processes define the channels, form the flow boundaries, or block the pores.^1,3^ Techniques such as photolithography, plasma treatment, and printing (inkjet and screen printing) are typically used.^10−15^ For example, Postulka et al. used a combination of wax printing and hot embossing to yield microfluidic channels on paper, in which the embossed areas formed the hydrophobic barriers that confined the fluid flow laterally.^15^ Furthermore, Li et al. developed microfluidic channels with inkjet printing and plasma treatments to generate a hydrophilic–hydrophobic contrast on a filter paper surface.^13^

Paper-based fluidic systems, however, suffer from relatively low pattern resolution, especially if they are highly porous, and the complexity of the channel design is usually limited.^1,16^ Therefore, there is a demand for diagnostic substrates to replace nitrocellulose and find other alternatives for standard paper substrates. Then again, with growing attention on printed electronics, the development of printed diagnostic devices requires integration of a fluidic channel with other components such as a display (to show the testing results), battery (as a power source), and antenna (for communication) in one platform (substrate). This challenge is addressed in the INNPAPER project, where we aim to develop all of the electronic components on one paper substrate. Although printing is commonly used in the production of paper-based microfluidic devices, related techniques are usually dedicated to printing hydrophobic polymers that form the channel boundaries. For example, Lamas-Ardisana et al. have produced microfluidic channels on chromatography paper by screen-printing barriers using UV-curable ink.^12^ We have also developed fluidic channels on nanopapers by inkjet printing a hydrophobic polymer that defined the channel.^17^ Though these methods are useful to produce paper-based fluidic channels, they cannot produce effectively integrated systems when applied on a printed electronic platform. Therefore, an alternative solution is considered by developing printable wicking materials to be deposited on the electronic platform and integrated with other components.

Recently, rod-coating of porous minerals, containing functionalized calcium carbonate (FCC) and various binders, was applied for developing wicking systems (see Jutila et al.^18−20^ and Koivunen et al.^21^). It was concluded that microfibrillated cellulose, applied as a binder, enabled faster wicking compared with synthetic alternatives such as latex, sodium silicate, and poly(vinyl alcohol). Besides, inkjet printing has been applied to define hydrophobic borders with alkyl ketene dimer (AKD) on the mineral coating, e.g., to provide an accurate outline of the fluidic channels.^20^ Finally, wicking materials printed on glass substrates have been reported using precipitated calcium carbonate (PCC) and a latex binder.^22^ Despite the recent reports, the advancement on adjusting formulations with both suitable wicking and required properties for large-scale printing has not been implemented. In this work, we developed stencil-printable wicking materials comprising calcium carbonate particles and micro- and nanocellulose binders. We demonstrate that the combination of nano- and microscaled fibrillated cellulose was necessary to achieve formulations with suitable wicking and printability. We further extended the printability of the wicking materials on flexible substrates (plastic and paper) using small amounts of additives as adhesives. This offers the possibility to develop printable fluidic systems for specific applications using multiple print passes, e.g., for printed electronics on paper substrates. Resistance to mechanical distortion is considered as one of the major requirements during such production processes, as well as subsequent robustness during handling in transport and end-use applications. By printing the wicking component, one can avoid the need for hydrophobic confinement, and the channel production can be scaled up within the roll-to-roll production of the printed electronic platform. As a demonstration, we show printed channels for chemical sensing of a nonspecific protein and glucose in clinically relevant ranges. To achieve fully printable sensing systems, the fluidic channels were printed on paper and functionalized with the given ligands using inkjet printing, demonstrating a simple and practical platform for multisensing. Thus, we show for the first time a robust platform that simultaneously provides optimal printability and adhesion on the substrate, as well as adjustable fluid flow properties for analyte wicking.

## Experimental Section

### Materials

Cellulose nanofibrils (CNF, 2.4 wt %) were produced from bleached Kraft birch fibers by microfluidization (M110P fluidizer, Microfluidics corp.) using six passes in 200 and 100 μm chambers under 2000 bar. High-consistency enzymatic fibrillation (HefCel) technology was used to produce fibrillated cellulose materials at a high consistency (19–23 wt %).^23,24^ Milled expanded perlite, a naturally occurring volcanic glass, was sourced from Omya Group (Omyasphere 120, Omya International AG, Oftringen, Switzerland). The paper substrate used was PowerCoat HD (a sized paper used for printed electronics), provided by Guarro Casas (Barcelona, Spain). Calcium carbonate (CaCO_3_) precipitated for analysis (EMSURE Reag. Ph. Eur.) with a particle size of approx. 14 μm and a surface area of 2.25 m^2^/g was purchased from Merck. Microscope glass slides (25 × 75 mm^2^) were from VWR International, and cationic starch (CS) (Raisamyl 150) was from Chemigate. Polyethyleneimine (PEI) 50% aqueous solution (*M*_w_ 600 000–1 000 000), poly(acrylic acid) (PAA), and propylene glycol (PG) were purchased from Sigma-Aldrich.

### Preparation of Stencil-Printable Pastes

To find the optimal formulation for fluid transport and printing, different compositions were prepared and tested assisted by computational modeling, which will be reported elsewhere. For the present work, due to the extent of the data, six ink formulations have been selected, as shown in Table 1. A wider analysis will be published in the future. First, CaCO_3_ was dispersed in deionized (DI) water (6 g). Then, the binders (CNF and/or HefCel) were added gradually to the CaCO_3_ paste together with perlite. The paste was mixed until homogeneity (10 g of total dry solids for each formulation). The pastes were named according to their composition. Ca–C, Ca–H, and Ca–CH denote systems containing CaCO_3_–CNF (95:5), CaCO_3_–HefCel (95:5), and CaCO_3_–CNF–HefCel (95:2.5:2.5), respectively. Additionally, CaP–C, CaP–H, and CaP–CH denote pastes containing CaCO_3_–perlite–CNF (85:10:5), CaCO_3_–perlite–HefCel (85:10:5), and CaCO_3_–perlite–CNF–HefCel (85:10:2.5:2.5), respectively.

**Table 1:** Formulations Used for Printed Channels According to Given Compositions (Particle-to-Binder on a Total 100 Parts Dry Basis) and Total Dry Solid Content

component	Ca–C	Ca–H	Ca–CH	CaP–C	CaP–H	CaP–CH
CaCO_3_	95	95	95	85	85	85
perlite				10	10	10
CNF	5		2.5	5		2.5
HefCel		5	2.5		5	2.5
dry solids (wt %)	27.5	56.6	37.0	27.5	56.6	37.0

### Rheology

The shear viscosity of the prepared pastes was measured with a dynamic rotational rheometer (Anton Paar MCR 302). Parallel plates (PP25) were used with a gap fixed at 1 mm. Shear rates from 100 to 1000 s^–1^ were used to measure changes in viscosity. All samples were measured five times at 23 °C.

### Stencil Printing of Fluidic Channels

The printability of the pastes was initially investigated by hand printing through a stencil on glass slides. A squeegee (RKS HT3 Soft, Seri-fantasy Oy, Helsinki, Finland) was used to transfer each paste through a plastic stencil (352 μm thickness), and linear channels (4 × 70 mm^2^) were formed on the substrates after removal of the stencil. Finally, the channels were dried overnight in a fume hood.

### Channel Thickness

#### Profilometry

The thicknesses of the printed channels were obtained with a profilometer (Dektak II Surface Profiler, Veeco Instruments Inc.). A 5000 μm scan length, a 2.5 μm stylus, and a 1.00 mg force were used during measurements. The average value of the thickness profile was calculated, and two replicates per sample were measured.

#### Confocal Imaging

The thickness profiles of the dried CaP–CH and Ca–CH channels were obtained with an optical confocal microscope (S Neox 3D Optical Profiler, Sensofar Metrology, Spain). An EPI 5× objective was used, and two replicates per sample were measured.

### Scanning Electron Microscopy (SEM)

The prepared channels were imaged with SEM to observe their morphology and porous structures. Besides, each paste component (CaCO_3_, perlite, CNF, and HefCel) was imaged separately. Before imaging, all of the samples were sputter-coated to deposit a 5 nm Au–Pd layer using a LEICA EM ACE600 sputter coater. Images of the channels were taken with a field emission microscope (Zeiss Sigma VP, Germany) at 1.5 kV.

### Wicking Tests

Vertical wicking experiments with a liquid supersource were studied in the prepared channels in a conditioned room at 21 °C and 60% relative humidity. Samples were placed upright with their free end suspended into a Petri dish (radius *r* = 2.7 cm, volume *V* = 25 cm^3^), and distilled water was added to wet the channel. A camera was used to record the wicking distance at 25 frames per second. At least three replicates were measured for each sample. To distinguish the wicking front line, the backside of the system was illuminated to produce a high contrast between the dry and wetted areas of the channel. An illustration of the test system can be seen in Figure S1. The propagation of the wicking front line as a function of time was analyzed with MATLAB R2019b (MathWorks) as follows. First, a rectangular region encompassing the channel was manually identified from the video. For one frame each second, a second-degree polynomial fit was subtracted from the graph of the median grayscale values calculated for each horizontal pixel row in the analyzed region to account for possible lighting variations along the channel. The wicking front was thereby distinguishable as a step-like change in the median grayscale graph, thus allowing the identification of its location from the mean of the Gaussian fit to the derivative of this plot (see Figure S2). A ruler was used to equate pixels to physical distances, *L*, which typically resulted in a resolution of roughly 10 pixels per 1 mm. The time “zero” frame was selected as the first frame when the wicking had visibly started (20 ms uncertainty).

### Printing on Paper Substrates and Adhesion

Channels were printed on the paper substrate (PowerCoat HD), suitable for various printing operations such as inkjet, flexo, and screen printing.^25^ The PowerCoat substrate includes a thin barrier layer, which gives water resistance and hydrophobicity. For simplicity, hereafter, we refer to PowerCoat as the “paper” substrate. The hydrophilic (water-containing) printed paste did not adhere adequately to the paper substrate. Therefore, further ancillary components were used as adhesives, specifically polyethyleneimine (PEI), cationic starch (CS), poly(acrylic acid) (PAA), and propylene glycol (PG). One approach was to coat a thin layer of the adhesive on paper before printing the channel. Namely, substrates were treated with PEI (5 wt % in EtOH), CS (1 wt % in H_2_O), or PAA (2 wt % in EtOH) solutions and left to dry. After drying, channels were printed with the CaP–CH and Ca–CH pastes on the pretreated papers. Another approach included the addition of an adhesive to the wet paste before printing the channels. Specifically, PG (2–5 wt % of the wet paste) was mixed into the Ca–CH paste and printed on the unmodified paper to form channels. Finally, the adhesion of the dried channels on the papers was evaluated by flexing the coating under bending and assessing the subsequent coating integrity by visual observation.

### Large-Scale Printing of the Fluidic Channels

CaP–CH with 2 wt % PG was printed with a semiautomatic stencil printer (EKRA E2, ASYS GROUP). A 100 μm thick stencil with several rectangular patterns (80 × 5 and 80 × 3 mm^2^) was used to produce channels on PET films and paper substrates. A stainless steel squeegee was used to spread the paste at a confining angle of 60° with a constant printing speed of 60 mm/s. To adjust the channel thickness, the gap between the stencil and squeegee was set to 300–600 μm.

### Protein and Glucose Sensing

Protein and glucose sensors were prepared by deposition (pipette) of the sensing reagents on Ca–CH channels printed on glass. The Biuret reagent was used for the detection of bovine serum albumin (BSA). The Biuret reagent for detecting protein was prepared by mixing 0.75% (w/v) of copper(II) sulfate pentahydrate (CuSO_4_·5H_2_O) and 2.25% (w/v) of sodium potassium tartrate in 50 mL of Milli-Q water.^26^ Then, 30 mL of 10% (w/v) NaOH was added while mixing. Finally, further Milli-Q water was added for a total volume of 100 mL. For protein sensing, BSA solutions of known concentrations (0, 25, 60, and 90 g/L) were applied to the channels. Then, 5 μL of the protein reagent was deposited on the sensing area. The detection of glucose was carried out by enzymatic reaction using glucose oxidase (GOx, 340 units) mixed with horseradish peroxidase (HRP, 136 units) in 10 mL of citrate buffer solution (pH 6)^27^ in the presence of 0.6 M potassium iodide (KI) (1:1 volume ratio).^10^ Glucose solutions of known concentrations (0, 2, 5.5, 7, 9, and 11 mM) were used with the given channels followed by the addition of 5 μL of the enzyme reagent to the sensing area. Multisensing assays were carried out with either water, BSA (25–50 g/L), or glucose (7–11 mM) solutions, as well as mixtures of BSA (25–50 g/L) and glucose (7–11 mM). In these cases, the Biuret reagent and enzyme system were separately applied on the opposite ends of the channel.

### Inkjet Printing of the Multisensing Assays

An inkjet printer (Dimatix Materials Printer, DMP-2831, Fujifilm) was used to form well-defined sensing areas and to produce multisensing assays on paper. Rectangular shaped (2 × 3 mm^2^) sensing areas were inkjet-printed on the Ca–CH channels (with 5 wt % PG) supported by the paper substrate. The protein and glucose reagents were filled in DMC-11610 cartridges (10 pL nominal drop volume) and printed with a drop spacing of 20 μm at a 5 kHz frequency, a 28 V jetting voltage, and a 3–5 in. H_2_O meniscus vacuum (∼7.5–12.4 mbar). Eight and 15 layers of protein and glucose reagents were printed on the sample, respectively. Cleaning was done with the “Purge 0.1 s” cleaning cycle at the beginning of the printing and after every 900 s or every 900 print bands.

### Image Analysis

The progression of sensing was recorded with a camera, and image analysis was performed. Specifically, the change in the grayscale color intensity of the sensing areas was evaluated with Adobe Photoshop 2021 software. To differentiate yellow and orange colors in the glucose assay, the images were converted to grayscale using a blue high contrast filter. By contrast, in the analysis of the protein, the images were converted to grayscale using an infrared filter to highlight the purple color. Then, the mean gray intensities, *I*, of the sensing areas were obtained using the histogram distribution. Finally, the intensity values were normalized by dividing the given intensity with the initial value, *I*/*I*_0_. On the one hand, in the protein assay, the sensing area was initially blue, and the grayscale intensity of this blue color was chosen as the initial value for the image analysis. On the other hand, in the glucose assay, the sensing area was colorless right after the reagent was deposited onto the assay; thus, this color of the wetted channel was chosen as the initial value for the glucose assay analysis.

## Results and Discussion

We first discuss the formulation of the pastes used to deposit fluidic channels on glass supports by stencil printing. The results on this idealized inert substrate are then used for the development of fluidic channels on paper. We study wicking effects, as well as detection of glucose and a protein (BSA) with the specifically purposed systems.

### Paste Formulation and Stencil Printing of Fluidic Channels on Glass

Pastes were produced from CaCO_3_, perlite, CNF, and HefCel, with each component displaying distinctive morphologies (Figure S3). The main component was the cubic-shaped precipitated CaCO_3_ (PCC).^22^ Note: we also tested ground calcium carbonate (GCC) but it was not considered further, given its irregular crystal shape and wide particle size distribution forming a tight, insufficiently permeable, packing. The second mineral, namely, expanded perlite (an amorphous volcanic glass showing platelet structures; Figure S3), was incorporated in some paste formulations. Perlite has been used as an adsorbent material^28^ and was added to improve water retention during printing.

Two binders, CNF and HefCel, were used to hold the minerals in the dry state. HefCel is composed of relatively large fibrils and fibril size distribution and has low water content and a paste-like consistency.^29^ Meanwhile, CNF has a high water-holding capacity and forms gel structures. We combined the two fibrillated celluloses to produce effective binding and achieve appropriate fluid flow properties combined with controlled water retention properties, which are not achievable in a paste when only a single cellulose component is used. The mass ratio mineral-to-binder was fixed at 95:5 according to their wettability and dimension (initial tests showed that high CaCO_3_ levels were necessary to obtain a suitably porous structure and effective permeation wicking performance).

CNF and perlite loading produced high viscosity under shear (see Figure S4a). By contrast, HefCel produced pastes with lower apparent viscosity, even at high solid content (note the lowest viscosity of paste Ca–H). This effect is explained by the larger fibril size distribution and variation in fibril network entanglement.^29^ The addition of perlite to the Ca–H paste at high solid content produced a solid-like behavior that prevented measurements. A greater shear-thinning behavior was noted for pastes that combined CNF and HefCel. Perlite had no significant influence on the viscosity of CaP–CH at low shear rates.

The prepared pastes were stencil-printed (Figure S4b) to produce channels on the glass slides (Figure S4c). The thicknesses, masses, and wicking characteristics of the printed channels are listed in Table 2. In addition, an exemplary thickness profile of the CaP–CH channel can be seen in Figure S5. The printability of the pastes varied based on the formulation, and their response to coating application shear followed that of the experimental flow analysis. The pastes that only contained HefCel binder were difficult to print due to the high solid content and the low shear-thinning effect. Specifically, the Ca–H paste was difficult to print since the thick paste required multiple sweeps over the stencil, which resulted in irregular edges (see Figure S4c). Further, the addition of perlite did not improve printability and the CaP–H formulation was the most difficult one to print. By contrast, CNF enhanced printability, but it held large volumes of water, making the paste a gel-like material. Specifically, the addition of CNF to CaCO_3_ particles produced a paste (Ca–C) that released excess water during printing. On the one hand, this suggests that the ionic content of the PCC (typically residual CaO/Ca(OH)_2_) acts to bridge adsorption between CNF and PCC, as shown similarly by Dimic-Misic et al. and Liu et al. for GCC and nanofibrillated cellulose.^30,31^ On the other hand, the addition of 10 wt % perlite to the Ca–C paste (CaP–C), known for its use in adsorbing cations,^32^ improved water retention and printability due to a more accessible porosity. Nevertheless, the best printability was obtained with a combination of both CNF and HefCel binders (Ca–CH and CaP–CH pastes). They also enabled channels with a better spatial resolution (Figure S4c) owing to the pronounced shear-thinning behavior, which was particularly suitable for stencil printing (Figure S4a).

**Table 2:** Effect of Paste Composition on the Characteristics of the Printed Channel^a^

			wicking constant (*D*)		wicking power (*p*)
channel	mass (mg)	thickness (μm)	*L* = *Dt*^0.5^	*L* = *Dt*^p^	*L* = *Dt*^*p*^
Ca–H	130 ± 0.6	352 ± 18	4.54	5.48	0.465
CaP–H	136 ± 39	372 ± 19	4.07	4.89	0.468
Ca–C	44.5 ± 3	200 ± 11	1.85	4.07	0.390
CaP–C	46.7 ± 2	207 ± 33	2.67	3.91	0.444
Ca–CH	65.0 ± 4	209 ± 10	3.14	4.42	0.449
CaP–CH	55.3 ± 6	201 ± 10	3.07	4.05	0.460

a The wicking constant (*D*) describes the capillary flow of the fluid related to the particulate pore structure of the channels. The *D* values are obtained by fitting the recorded flow distances according to either the Lucas–Washburn (L–W) model (function of *t*^0.5^) or as the power law function (function of *t*^*p*^).

As shown in Table 1, the solid content of the wet pastes containing HefCel was higher (56.6 wt %) compared to that based on CNF (27.5 wt %). This is because the water content of the original HefCel material (19–23 wt % consistency) was less than that of CNF (2.4 wt % consistency). In an attempt to improve the printability of Ca–H and CaP–H, various amounts of water were added to the pastes. The addition of 9.33 g of water (for 37 wt % solid content in wet pastes) turned pastes into liquid suspensions, which were not suitable for printing. With the addition of 1 g of water, a paste containing 53 wt % solids was obtained, yet it was still difficult to print due to poor water retention. During printing, excess water run-off caused smearing of the channel pattern. Even with a small amount of water (0.5 g), the pastes with 55 wt % solids could not retain water properly, leading to poor printing (see Figure S6). Therefore, it was concluded that the printability of these pastes could not be improved directly by adjusting their water content only. Evidently, high water-holding capacity is needed for paste printability and therefore the use of CNF was shown to be crucial to enable the printing process.

**Figure 1:**
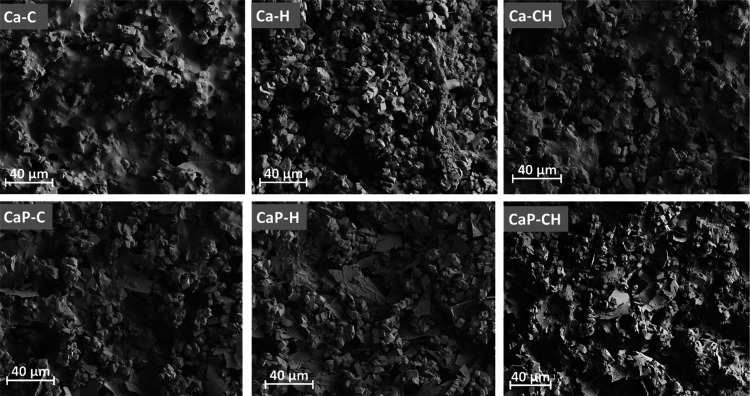
SEM images of the top view of the printed channels. Ca–C, Ca–H, and Ca–CH denote pastes containing CaCO_3_–CNF (95:5), CaCO_3_–HefCel (95:5), and CaCO_3_–CNF–HefCel (95:2.5:2.5), respectively. Additionally, CaP–C, CaP–H, and CaP–CH denote pastes containing CaCO_3_–perlite–CNF (85:10:5), CaCO_3_–perlite–HefCel (85:10:5), and CaCO_3_–perlite–CNF–HefCel (85:10:2.5:2.5), respectively. Scale bar: 40 μm.

Figure 1 shows the SEM images of the dried printed channels. Additionally, the SEM images of the paste components and the cross-sectional images can be seen in Figures S3 and S7, respectively. It can be observed that both CNF and HefCel have effectively connected the CaCO_3_ particles to form networked structures. Specifically, CNF formed a tight network between the particles, as observed in Ca–C. HefCel in Ca–H formed a network that was not as tightly formed, resulting in a looser packing. The SEM images highlight this difference between the two binders, i.e., one consisting of nanosized fibrils (CNF) and the other with larger nano/microfibrils, and their influence on interparticle pore connectivity (term used to describe the number of nearest-neighbor pores that can be accessed from a single pore during permeation), which resulted in different fluid permeabilities. The combination of HefCel and CNF (Ca–CH), thus, resulted in channels with intermediate pore connectivity, and the addition of platelet-structured perlite slightly changed the channel network structure. Since the connected porous structure of the channels significantly influenced the fluid flow property, we discuss next the effect of structure in more detail later.

### Fluid Wicking

**Figure 2:**
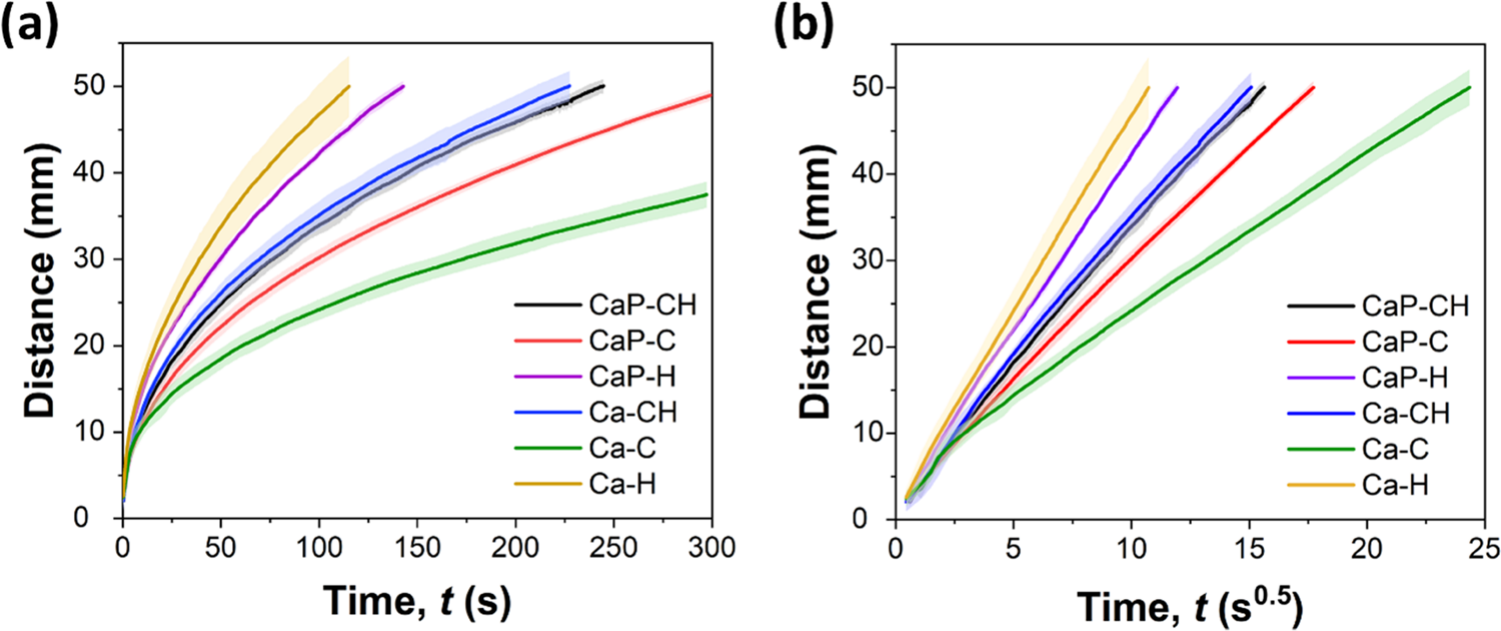
Wicking front line in channels: (a) the raw data and (b) data adjusted to the Lucas–Washburn equation. Curves represent mean ± standard deviation (shading) from three samples.

The fluidic channels were investigated in vertical wicking experiments with water (see the flow curves in Figure 2a). The shadowed areas in the flow curves represent the dispersion of data and indicate repeatable results for the printed channels.

Generally, porous media transport fluids by wicking according to surface wetting and capillary action, which, at equilibrium flow, can be followed by the Lucas–Washburn’s (L–W) model^33,34^ that relates the distance of liquid flow (*L*) with respect to the square root of time
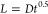
1where *t* is the fluid permeation time and *D* is the wicking constant related to the interparticle capillary and intraparticle pore structure.^35^ The flow distance measured for all of the channels was fitted according to the L–W model (eq 1) and presented as a function of *t*^0.5^ (Figure 2b; the derived wicking constant (*D*) is listed in Table 2). Figure 2 shows that Ca–H achieved the fastest flow, reaching 4 cm in 70 s, while Ca–C demonstrated the slowest flow (4 cm in 350 s). The *D* values (Table 2) for Ca–H and Ca–C correlate with the observed structure of the channels in SEM micrographs (Figure 1), i.e., Ca–H is more loosely packed compared to Ca–C, which enhanced the fluid flow. Alternatively, the channels made of both CNF and HefCel (Ca–CH) wicked water along 4 cm in almost 130 s, which resembled the intermediate *D* value and intraparticle network observed in the SEM image. According to the *D* values, perlite exerted a minor effect on the wicking properties of the channels containing HefCel and combined binders (CaP–H, CaP–CH). In contrast, a noticeable wicking improvement was achieved with the addition of perlite in a channel containing CNF binder (CaP–C). This may be explained by the platelet-like structure of perlite with various sizes, which positioned among CaCO_3_ particles and CNF, thus increasing interparticle pores within the network^36^ (Figure 1). The wicking properties of our channels with the optimum composition (Ca–CH, CaP–CH) demonstrate a clear improvement over previously reported channels containing microfibrillated cellulose and FCC (4 cm water wicking in 500 s).^18^ Furthermore, our printed channels wicked fluid almost similarly to filter paper (Whatman 3, 3 × 70 mm^2^, 390 μm thickness), which wicked 4 cm of water in 100 s.

It should be noted that when we tested other particles such as ground calcium carbonate (GCC), we did not obtain suitable wicking properties, given its more regular particle shape and insufficient permeability. Testing silicate-based minerals, particularly laminate types, such as kaolinite and montmorillonite, was considered inappropriate due to both their organo-intercalative reactive nature causing potential reaction with bioreagents and enzymes, and impermeable, highly tortuous packing structures. In addition, it was observed that applying inert silica particles and fumed silica, in turn, formed a tightly packed structure that significantly slowed down the wicking properties. We also investigated the combination of PCC with silica particles and similarly observed decreasing in the wicking properties. From these efforts, we found that crystal-agglomerate PCC particles, with close to monodisperse micrometer size, can create a porous network that is readily bound together with nano/microcellulose and serve the purpose to wick the fluid effectively.

The liquid flowing through a channel tends to evaporate from the porous surface, and hence additional phenomena affect the wicking: the wicking rate is usually hindered and departs from the L–W model.^35^ Therefore, to define the nature of the flow system more precisely, the experimental data were fitted to a power law function
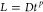
2where *D* is the wicking constant, *t* is the wicking time, and *p* is the wicking rate power law index, which is the slope of the flow curve in the logarithmic scale.^18^ By fitting the flow curves to eq 2 (Figure S8b), the exponent *p*, 0.390–0.468, clearly deviated from the L–W value (Table 2). This deviation is mostly caused by the inherent properties of the formed water retaining porous structure, where particle pores no longer contribute to the permeability but trap liquid. The systems that followed the L–W model were related to viscous permeation flow through the bulk pore network structure of the porous medium such that the constraining resistance to the wetting force at the liquid front was the sample bulk permeability. The trend in *p* values <0.5 reveals other effects related to pore wetting selectivity delay during acceleration at the wetting front, affecting mainly larger pores,^37,38^ and absorbing pores lacking further exit connectivity; the so-called ink-bottle pores^39^ can be ranked as Ca–H (0.465) > Ca–CH (0.449) > Ca–C (0.390), and the addition of perlite slightly increased *p* in all of the cases.

To summarize, the effect of each component, particle type, and binder has a significant effect on printability and flow properties. CNF improved printability given its shear-thinning effect but hindered fluid flow. Meanwhile, HefCel increased wicking but was rather challenging for printing, given its low water-holding capacity. A high ratio of CaCO_3_ particles to binder (95:5) was necessary to obtain a suitable porous structure and wicking (a slight increase in binder ratio, to 10 or 15%, affected wicking negatively; Figure S9). Perlite improved printability due to better water retention. Additionally, it improved wicking when CNF was used, while slightly hindered wicking was observed with HefCel. An optimal formulation, both for wicking and for printability, was found in the CaP–CH and Ca–CH pastes, which were applied further for developing the sensing platforms.

### Channel on Paper Substrates and Printing Scale-Up

**Figure 3:**
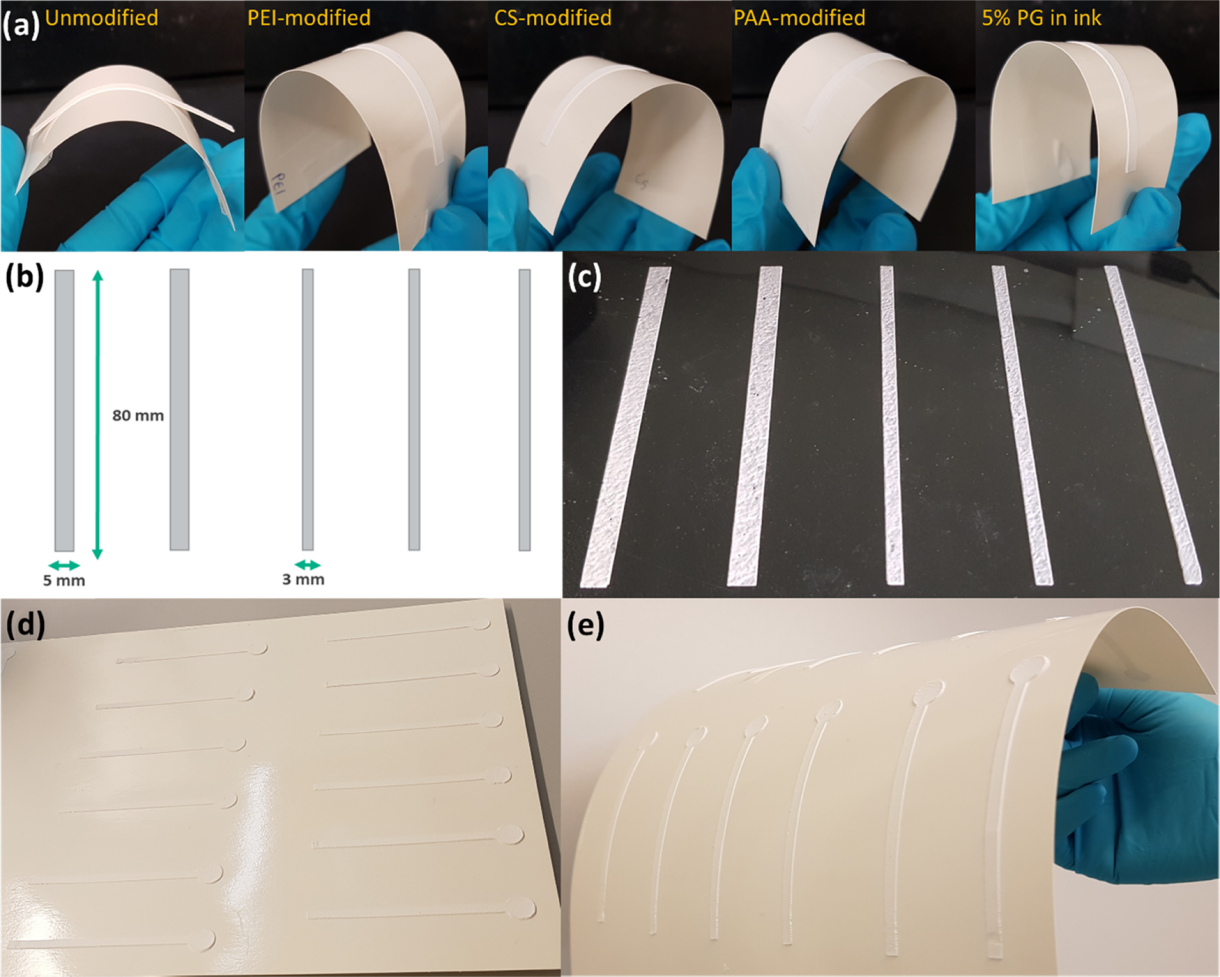
(a) Hand-printed channels on a paper substrate and improved adhesion were obtained with adhesives. (b) Stencil design for an industrial-scale stencil printer: channel width 3 or 5 mm and length 80 mm. (c) Channels on a PET film printed with the semi-automatic stencil printer (300 μm gap between the stencil and squeegee) using CaP–CH (+2% PG) paste. (d) and (e) Channels printed on paper substrate showing alternative design pattern with circular sample addition area.

The paper used herein was sized [water contact angle (WCA) of 94°, surface energy of 35 mN/m], which is desirable for applications that demand water resistance. This applies not only to packaging materials but, in the context of the current application, also beneficial for print quality and to prevent any effect from wicking by the substrate. However, such high WCA also made the task of achieving adhesion of the printed channels on the surface challenging. This latter effect was minimized by treatment with PEI, CS, PAA, and PG before printing the channels (Figure 3a). A thin layer of PEI, CS, and PAA was applied on the paper to enhance the adhesion and to produce channels that resisted mechanical bending. Also, the addition of PG in the paste, before printing, significantly improved adhesion. Applying PG as an adhesive provided a facile alternative for printing on a larger scale, even on PET films. It should be noted that charged polymers may influence the interaction of the solid phase with proteins and other analytes. However, here, the use of various cases as adhesives is demonstrated to show alternatives. Ultimately, the selection of the adhesive components should be based on targeted application, and in this work, we demonstrated the large-scale printing of our fluidic channels using PG to minimize any possible interaction with the bioreagents.

Multiple channels were printed using the CaP–CH paste, which was shown to be most suitable for large-scale printing (see stencil design and the printed channels in Figure 3b,c, respectively). The produced channels accurately reproduced the stencil pattern. The possibility to employ printing technology with the prepared pastes provides an opportunity for large-scale production of the channels and enables alternative designs. For example, an alternate design pattern comprising a linear fluidic channel with a circular sample addition area is presented in Figure 3d,e.

### Protein and Glucose Detection

Next, we demonstrate the detection of nonspecific protein and glucose in printed Ca–CH channels. Protein and glucose assays are common tests used in the diagnosis of several disorders and diseases. The normal level of protein in blood serum is 60–80 g/L.^40^ For instance, high total protein levels can indicate certain ill-health conditions, such as chronic kidney and liver disease, while low levels can indicate congestive heart failure, celiac disease, or liver and kidney diseases.^41^ The normal level of glucose in blood plasma after fasting is below 5.5 mM.^42^ Higher values indicate hyperglycemia, which is common for people with diabetes. Namely, people with prediabetic and diabetic conditions show blood plasma glucose levels of 5.5–6.9 mM and higher than 7 mM, respectively.^42^

**Figure 4:**
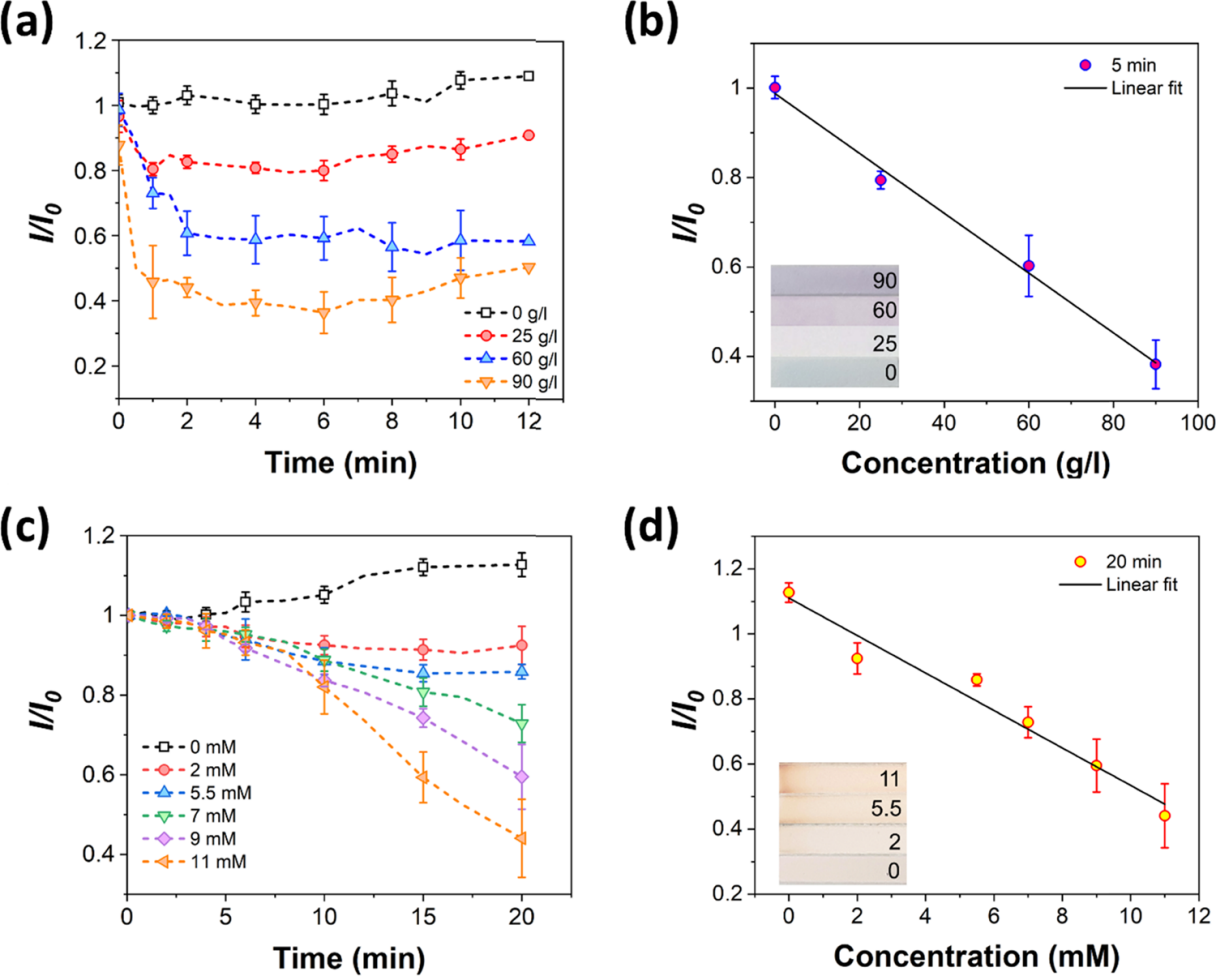
Protein and glucose sensing on the printed channels: (a) normalized color intensity on the protein-sensing area at different BSA concentrations, (b) calibration curve for the protein assay (inset: color on the sensing areas at different concentrations, unit: g/L), (c) normalized color intensity on the glucose-sensing area at different concentrations, and (d) calibration curve for the glucose assay (inset: color on the sensing areas at different concentrations, unit: mM). Curves represent mean ± standard deviation from three parallel samples.

We measured clinically relevant concentrations of glucose and bovine serum albumin (BSA) with the prepared fluidic systems. First, protein and glucose assays were prepared on separate channels and tested with 0–90 g/L BSA and 0–11 mM glucose. The sensing results are shown in Figure 4, where the normalized color intensities as a function of time for different concentrations are presented. The normalization was done by dividing the measured grayscale color intensity values with an initial grayscale intensity value (*I*/*I*_0_) (see the Experimental Section). Nonspecific detection of BSA was tested using the Biuret reagent. In the Biuret reaction, cupric ions (Cu^2+^) form a complex with a substance containing more than two peptide bonds in an alkaline solution, causing the reagent to change color from blue to purple.^26,43^ The deeper the purple color, the greater is the degree of complexation with Cu^2+^. When the images were changed to grayscale, the sensing area in the protein assay was initially light gray-colored and changed to dark black in the presence of BSA. The measured decrease in intensity indicated the presence of proteins. The reference channel exposed only to water showed no change in intensity (Figure 4a). Only a minor increase was observed after approx. 8 min, which was caused by the drying of the channel, which made the color lighter. When BSA was present, a rapid and evident decrease in color intensity (darker channel color) was observed, and a stable color was obtained after a few minutes. The effect of protein content was, therefore, clearly apparent (Figure 4a), and a calibration curve for the protein assay (Figure 4b) showed a linear dependence between *I*/*I*_0_ and BSA concentration. It should be noted that there could be an effect in the colorimetric response if human samples such as blood plasma would be tested. The example test demonstrated here is not to be considered an absolute measure design but illustrative how the designed structure might work to provide the basis for a test.

The detection of glucose was tested using a GOx/HRP/KI-based reagent. This type of glucose sensing is based on the enzymatic oxidation of glucose by GOx in an aqueous matrix in the presence of oxygen that forms gluconic acid and hydrogen peroxide. The HRP reduces the formed hydrogen peroxide to water and consequently, iodide is oxidized to iodine, forming a dark color.^10^ Initially, deposition of the enzyme system changed the sensing area from colorless to yellow and then eventually to brownish orange. Like the protein assay, the images of the glucose assay were changed to grayscale, and a decrease in intensity indicated the presence of glucose. The normalized color intensities on the glucose-sensing assay can be seen in Figure 4c. The reference sample showed only an increase in intensity due to the drying of the channel. By contrast, a decrease in color intensity was observed with the samples containing glucose, indicating oxidation of iodide into iodine. The development of color was slower compared to the protein assay, and the analysis of the color change was stopped after 20 min. The glucose sensor also showed a linear dependence of the color intensity to sample concentration (Figure 4d).

Color analysis was also performed for assays prepared on cut filter paper strips to represent the current performance of typical uncoated paper diagnostics, and the results of the normalized color intensity are provided in Figure S10. Compared to the printed channels, similar performance was recorded in the glucose sensing with the filter paper and so follows the use of such test systems already today. However, the protein sensor showed only qualitative responses on filter paper. Hence, the protein sensors could detect the presence of BSA (sensors changed from blue to purple) but quantitative sensing could not be obtained since the difference between concentrations could not be distinguished (Figure S10a). It is reasonable also to assume that the sensing reaction occurred more effectively in the printed channel due to its higher alkalinity caused by the CaCO_3_ compared with mineral-free filter paper.

### Simultaneous Detection of Protein and Glucose

**Figure 5:**
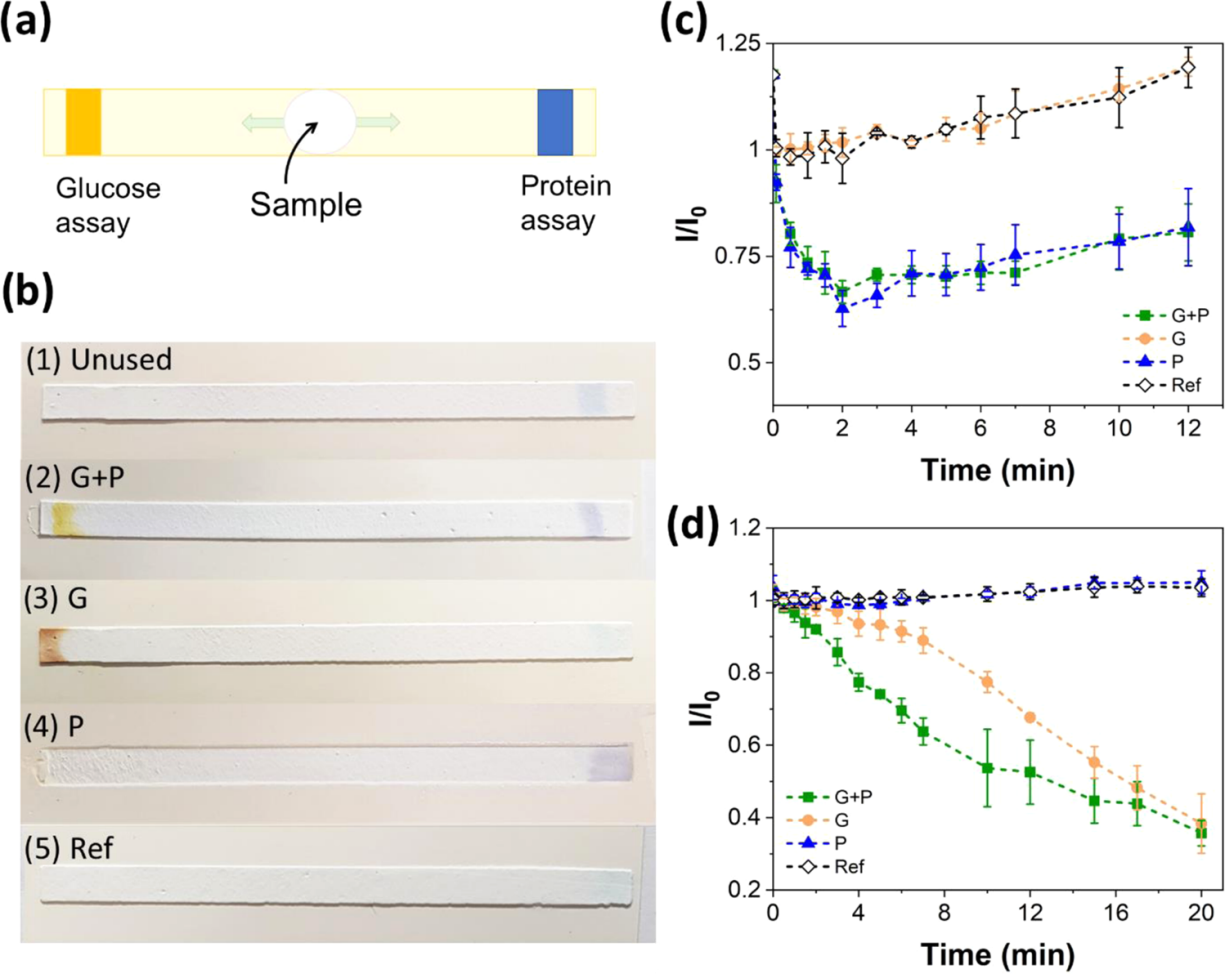
Multisensing assays: (a) schematic illustration and (b) inkjet-printed multisensing assays on paper substrates, showing color responses with different samples: (1) untested channel, (2) 7 mM glucose and 50 g/L BSA, (3) 7 mM glucose, (4) 50 g/L BSA, and (5) Milli-Q-water. Image analysis was used to obtain the sensing curves for protein and glucose sensing. (c) Normalized color intensities at the protein-sensing areas (right side) with the different samples: (G + P) 11 mM glucose and 25 g/L BSA, (G) 11 mM glucose, (P) 25 g/L BSA, and (Ref) Milli-Q-water. (d) Normalized color intensities at the glucose-sensing areas (left side) with the different samples: (G + P) 11 mM glucose and 25 g/L BSA, (G) 11 mM glucose, (P) 25 g/L BSA, and (Ref) Milli-Q-water. Curves represent mean ± standard deviation from three parallel samples.

Protein and glucose assays were applied to the channels printed on the sized paper to form a multisensing system. Initially, protein and glucose-sensing reagents were inkjet-printed on the opposite ends of the channels, and then BSA and/or glucose solutions were introduced at the center (Figure 5a). The color response in the inkjet-printed assays with different samples can be seen in Figure 5b. Before applying analyte solutions, the protein-sensing area is seen as light blue at the right end of the channel, and the glucose-sensitive area is colorless at the left end.

To study more carefully the color changes in multisensing, image analysis was performed. The normalized color intensities at the protein- and glucose-sensing areas with the different samples are shown in Figure 5c,d, respectively. The channels exposed to both glucose and protein (2, G + P) changed color at both ends of the channel: colorless to yellow in the glucose assay and blue to purple in the protein assay. Thus, a decrease in intensity was observed in both protein and glucose assays (Figure 5c,d). The channel exposed to glucose only (3, G) changed color in the glucose assay (Figure 5d) but the protein assay did not react to change color but lost its blue coloration gradually (Figure 5c). Interestingly, this assay had a slightly different color when compared to the channel exposed to both glucose and BSA (see Figure 5b). If BSA was present together with glucose (2, G + P), the color changed to yellow and did not turn as dark as the channel exposed to glucose only (3, G). However, after some time, the color intensities approached similar values (Figure 5d). It is possible that BSA acts as a stabilizer for the glucose reagent, affecting the color change. Indeed, it has been reported that BSA can bind to enzymes and act as a stabilizer.^44,45^ Supporting this explanation, BSA alone did not react with the glucose reagent, which can be seen in the channel exposed to only protein (4, P). This channel showed a color change in the protein assay (Figure 5c) but the glucose assay did not react (Figure 5d). This means that BSA does not cause oxidation in the glucose-sensitive reagent but possibly affects the activity of the GOx. In addition, the color response in the protein assay was very similar to the channel exposed to both analytes (2, G + P) (Figure 5b,c). Finally, the channel exposed to water alone (5, Ref) did not show significant color changes (Figure 5b–d).

Multisensing assays were also prepared on printed Ca–CH channels (using glass substrates) and filter paper by drop-casting the reagents with a micropipette (Figure S11b,c). Evidently, more controlled dimensions could be obtained for the sensing areas *via* inkjet printing. Besides, the channels prepared on filter paper experienced severe bending after wetting due to fiber swelling. Additionally, the detection behavior of the multisensing assays on printed channels was compared with assays prepared on filter paper strips (Figure S12). In protein sensing, there was a clear difference between samples with and without BSA. However, a slight variation in the color response between the reference and glucose sample was observed. Nonetheless, glucose sensing on filter paper was confirmed to be successful. Interestingly, the color difference between the G + P and G samples was not as noticeable in the glucose assay on filter paper as on the printed channel (Figure S11c). Therefore, the change in the normalized intensity of the assays on filter paper, as seen in the sensing curves, followed each other more closely (Figure S12b).

## Conclusions

Stencil-printable pastes were developed from minerals and cellulose binders to form fluidic channels that were easily printed on glass, plastic, and paper supports. The use of nanoscale cellulose fibrils ensured optimal printability, and the microscale fibrils guaranteed good wicking properties, exceeding previously reported flow rates. Various adhesives were used to improve the attachment of the channels to hydrophobic substrates, the most effective being propylene glycol, which enabled direct printing of the formulation on the desired substrate without pretreatments. Thus, the proposed fluidic systems can be used in applications requiring resistance against failure when subjected to bending, for example, in flexible diagnostics or electronics. The possibility to print the wicking component of the channel enables the formation of more complex fluidic systems and more tunable channel designs compared to the traditional paper-based fluidic systems. We demonstrated the channel sensitivity to protein and glucose in clinically relevant ranges, which opens the possibility for such fluidic channels as biosensors, for example, in drug analysis, disease diagnostics, environmental monitoring, or food quality control.

## Supporting Information Available

Additional information is provided online and includes illustrations of the setup for vertical wicking experiments and additional analyses of experimental data; SEM images of the channel components and channel cross sections; viscosities of the pastes as a function of shear rate; images of the channels printed on glass substrates showing the pattern quality of each paste; confocal image and shape profile of a printed channel; additional flow curves of channels with a higher binder ratio; and results of protein and glucose sensors on filter paper (PDF)
