# Supplementary material for: Bicomponent Cellulose Fibrils and Minerals Afford Wicking Channels Stencil-Printed on Paper for Rapid and Reliable Fluidic Platforms
Source: ACS Appl Polym Mater. 2021 Oct 5;3(11):5536–46. doi: 10.1021/acsapm.1c00856 (PMC8593863; doi:10.1021/acsapm.1c00856)
Supplement: Supplementary file 1 — ap1c00856_si_001.pdf [file ap1c00856_si_001.pdf]

SUPPORTING INFORMATION

Bicomponent cellulose fibrils and minerals afford wicking channels stencil-printed on paper for rapid and reliable fluidic platforms

Katariina Solin[1,2], Maryam Borghei[1,], Monireh Imani[1], Tero Kämäräinen[1], Kaisa Kiri[3], Tapio Mäkelä[3], Alexey Khakalo[2], Hannes Orelma[2], Patrick A. C. Gane[1], and Orlando J. Rojas[1,4,*]*

[1] Department of Bioproducts and Biosystems, School of Chemical Engineering, Aalto University, Vuorimiehentie 1, FI-00076, Espoo, Finland

[2] VTT Technical Research Centre of Finland Ltd., Functional cellulose, Tietotie 4E, FI-02044 Espoo, Finland

[3] VTT Technical Research Centre of Finland Ltd., Micronova, Tietotie 3, FI-02150 Espoo, Finland

[4] The Bioproducts Institute, Departments of Chemical and Biological Engineering, Chemistry and Wood Science, University of British Columbia, 2360 East Mall, Vancouver, BC, V6T 1Z4 Canada

Corresponding authors:

*E-mail: orlando.rojas@ubc.ca

*E-mail: maryam.borghei@aalto.fi

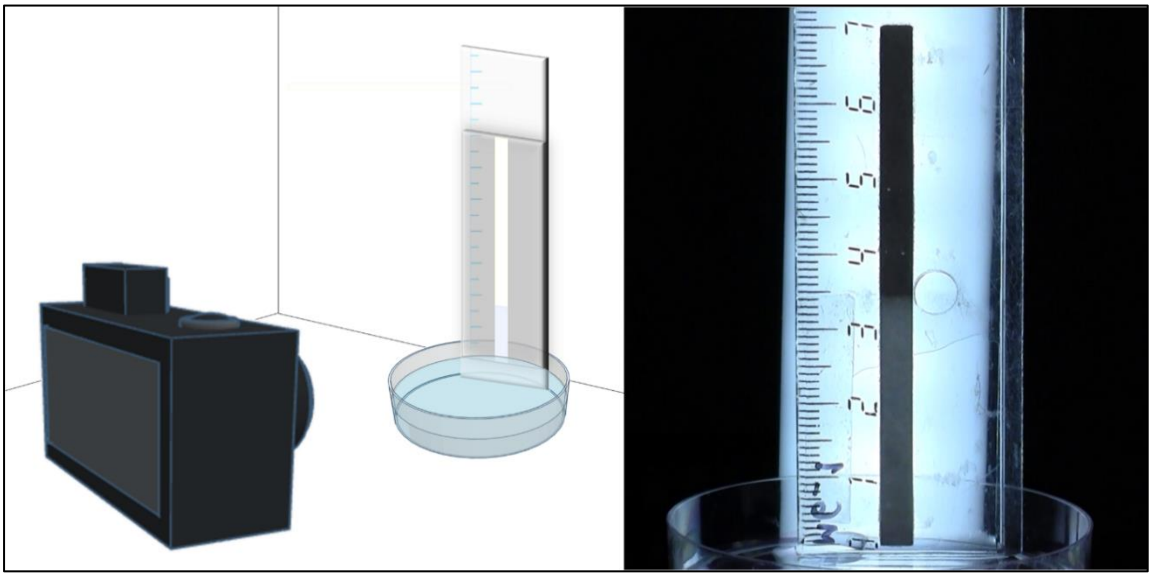

Figure S1. Set-up for vertical wicking. The positioning of a light source behind channels produced better contrast between the wet and dry channel and the wicking front-line could be readily distinguished.

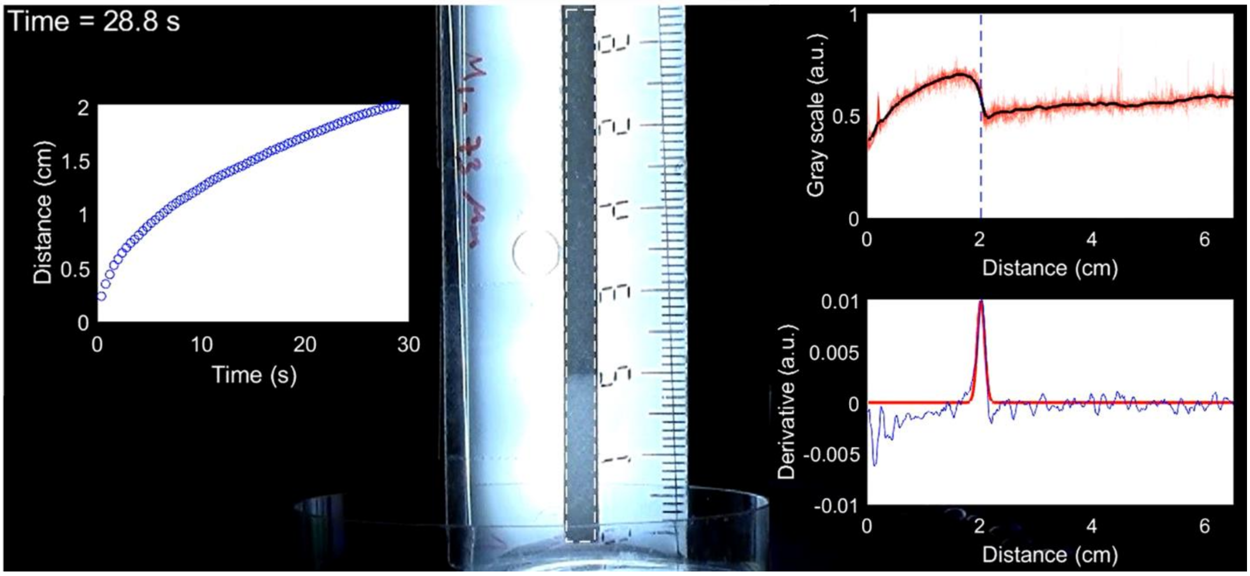

Figure S2. The wicking distance was analyzed as a function of time (left) by locating the step-like greyscale color change associated with the wicking front (top right, median value in black) within the white dashed box shown in the middle by using a Gaussian fit to the derivative of the median greyscale value (bottom right, red line).

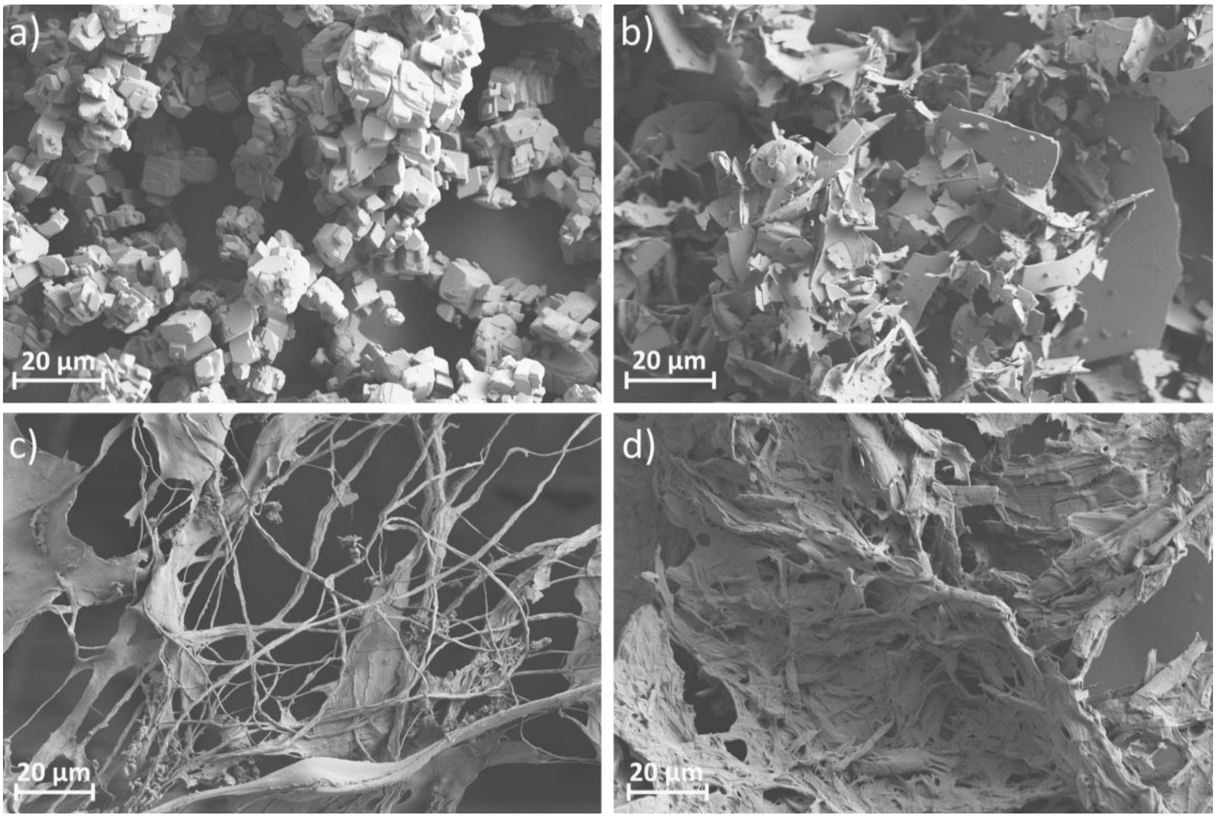

Figure S3. SEM images of the channel components: a) $CaCO_3$, b) Perlite, c) CNF, and d) HefCel.

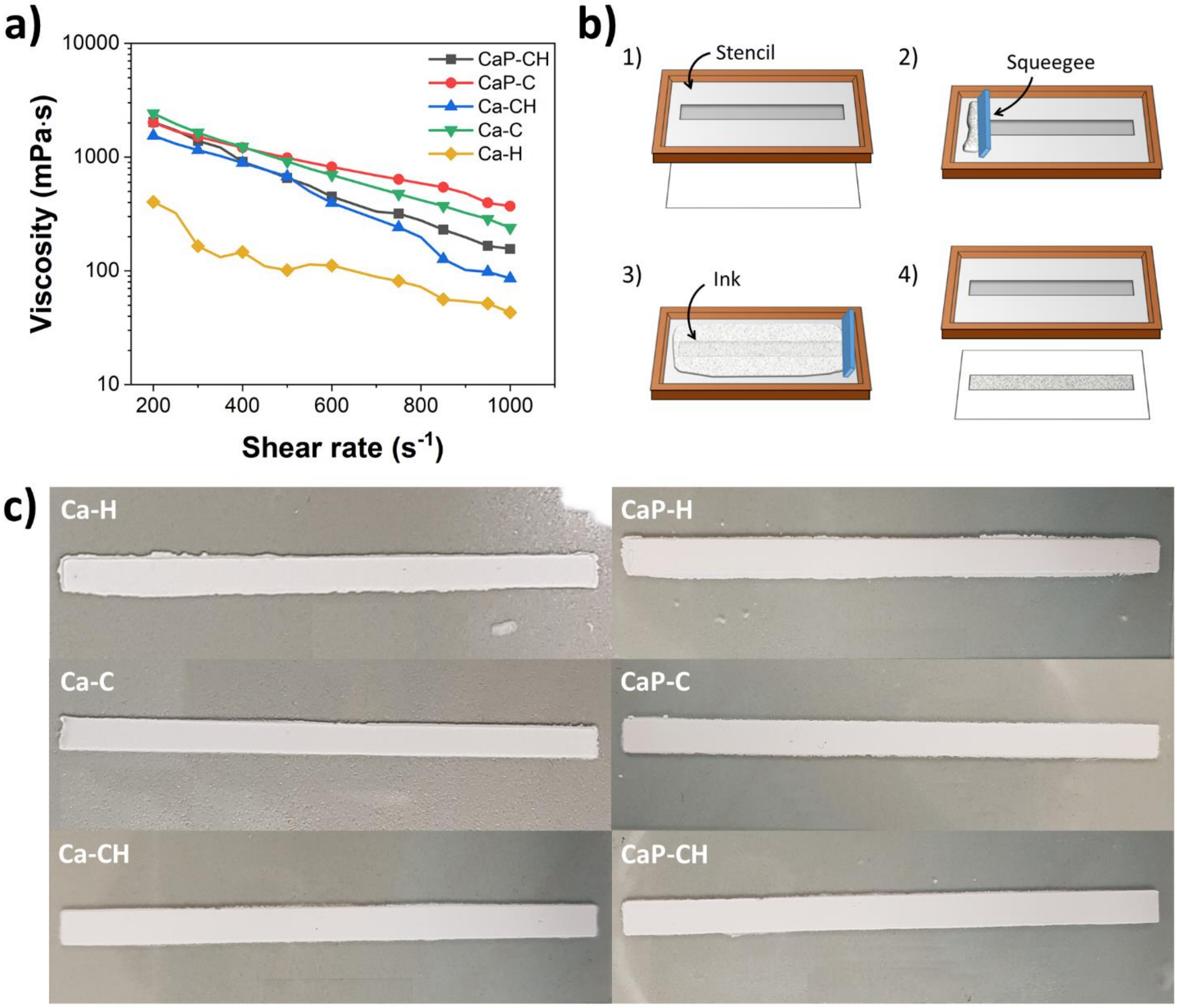

Figure S4. a) Viscosities of the pastes as a function of shear rate. b) Schematic illustration of the stencil-printing: 1) a stencil is placed on top of a substrate, 2–3) the paste is deposited near the desired pattern area and a squeegee is used to push the material through the stencil, 4) the stencil is lifted and the printed pattern is formed on the substrate. c) Images of the printed channels (width 4 mm, length 70 mm) on glass substrates showing the pattern quality of each paste.

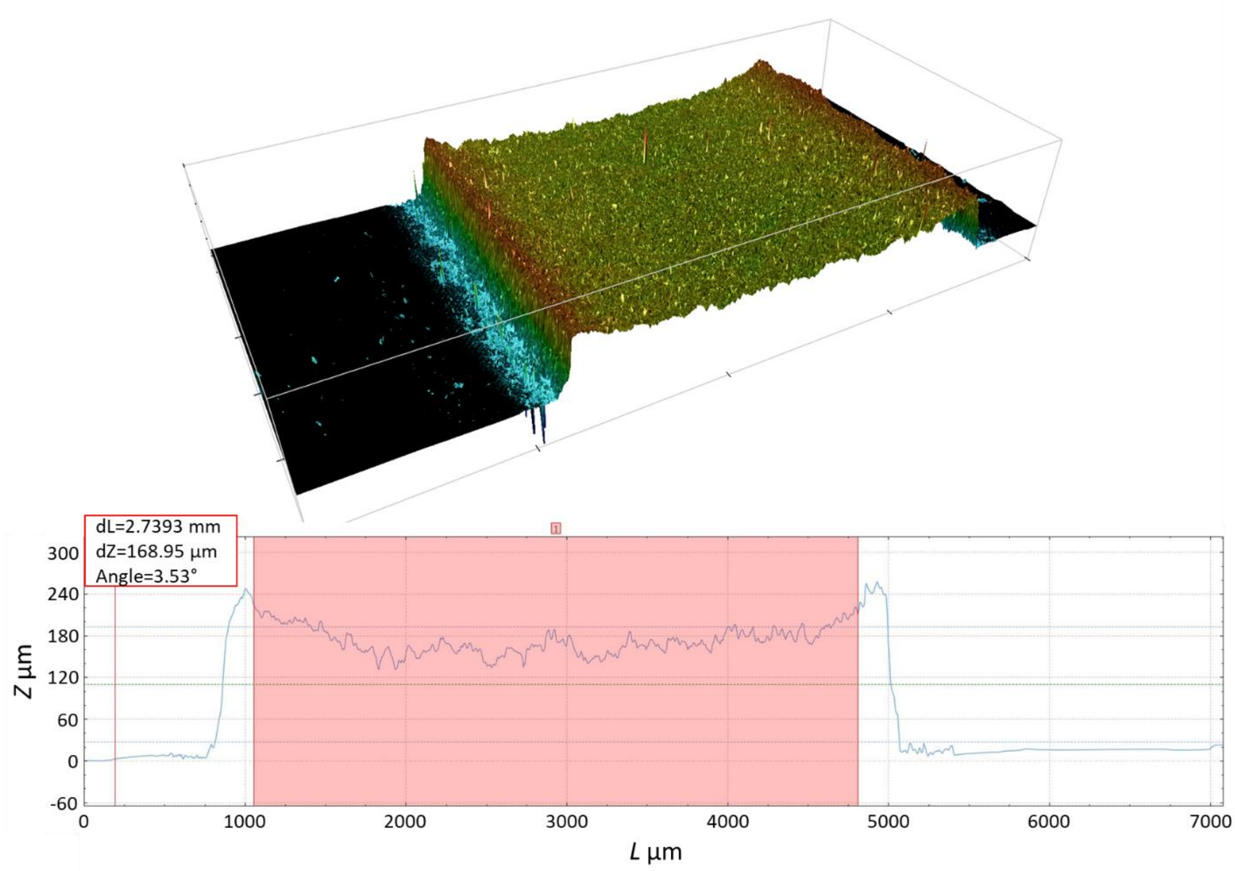

Figure S5. Confocal image and shape profile of the printed CaP-CH channel. The thickness profiles of the channels were concave-shaped, which can be seen from confocal images. This shape was caused when the stencil was lifted, and the channel edges moved upwards along with the stencil.

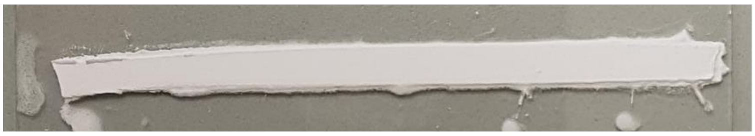

Figure S6. Adjustment of the solids contents of the pastes with HefCel (Ca-H and CaP-H) did not improve printability due to poor water retention.

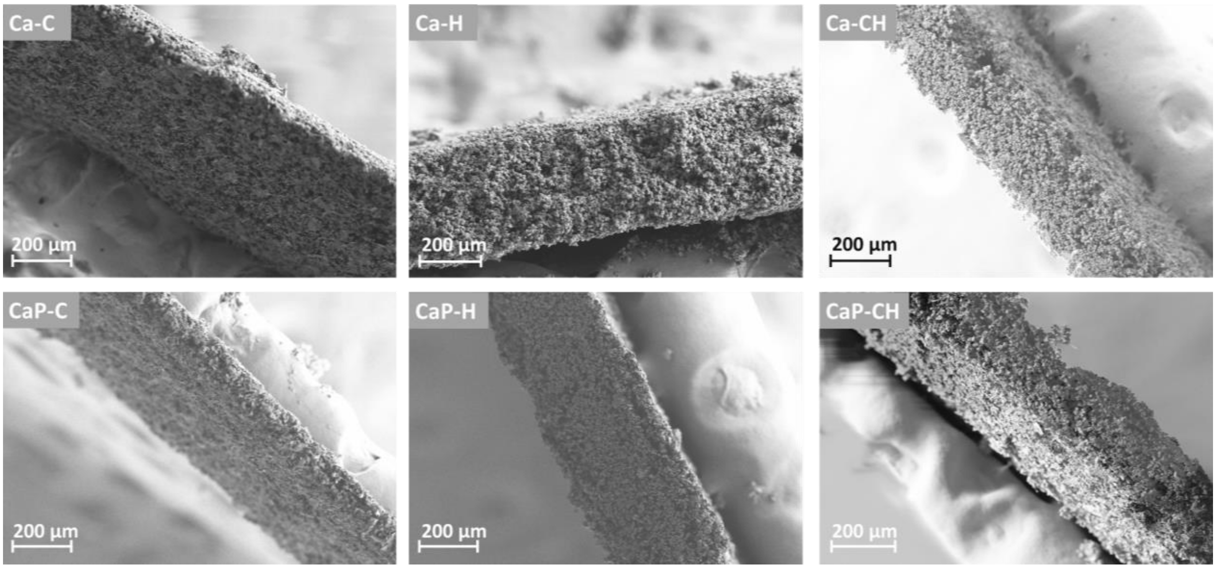

Figure S7. SEM images of the channel cross-sections.

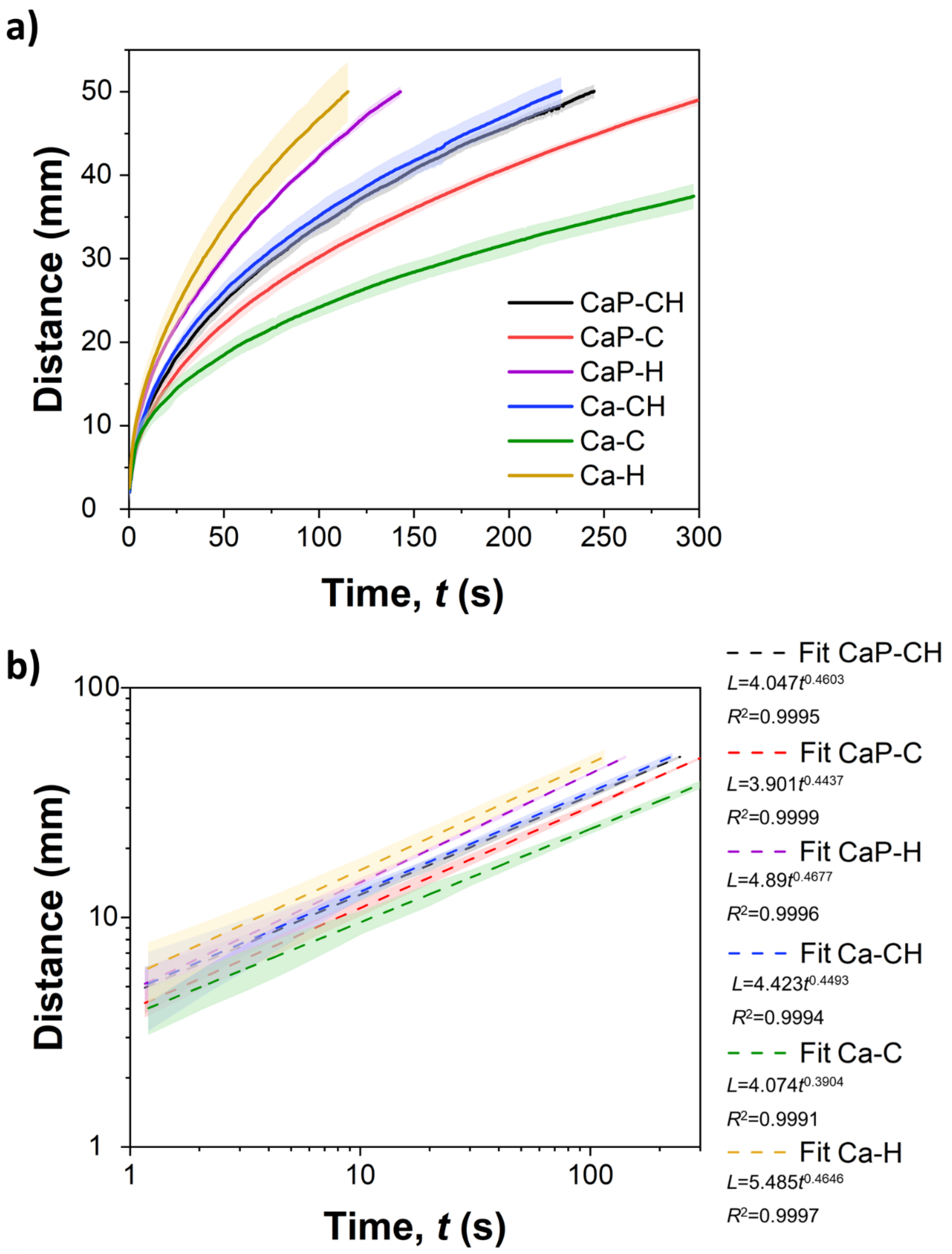

Figure S8. Flow curves: a) the raw data and b) the data fitted to power law function. Curves represent mean ± standard deviation from three parallel samples.

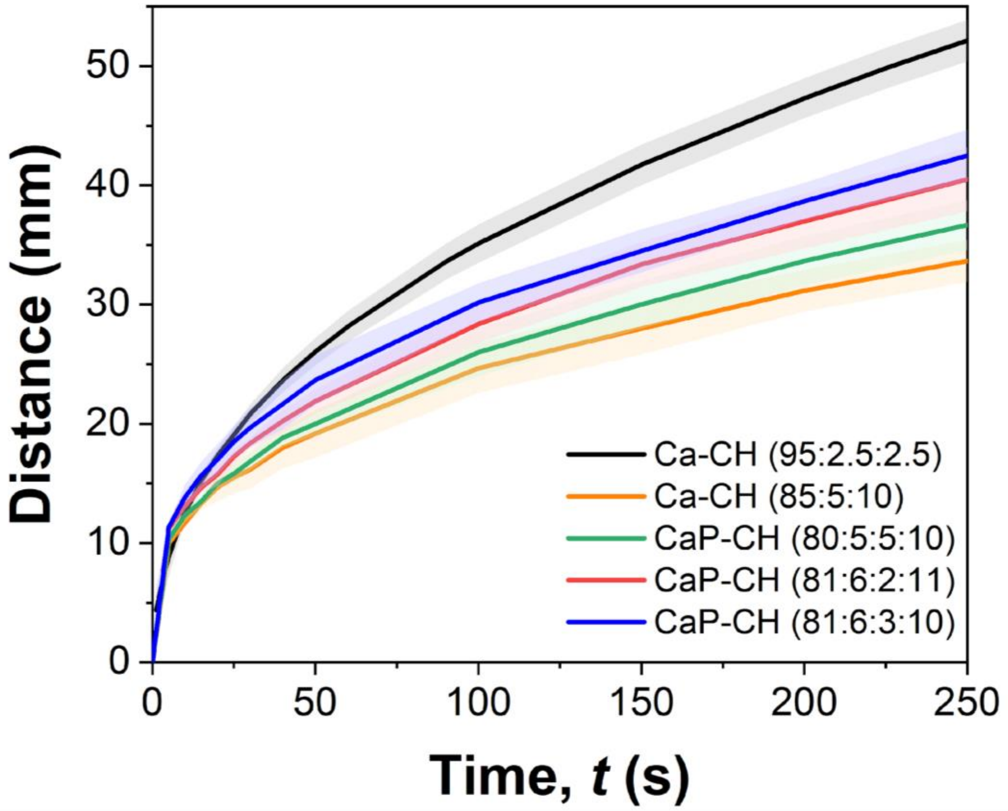

Figure S9. The high amount of mineral (black profile) was best for the flow properties, whereas a higher binder ratio reduced the flow rate. Curves represent mean ± standard deviation from three parallel samples.

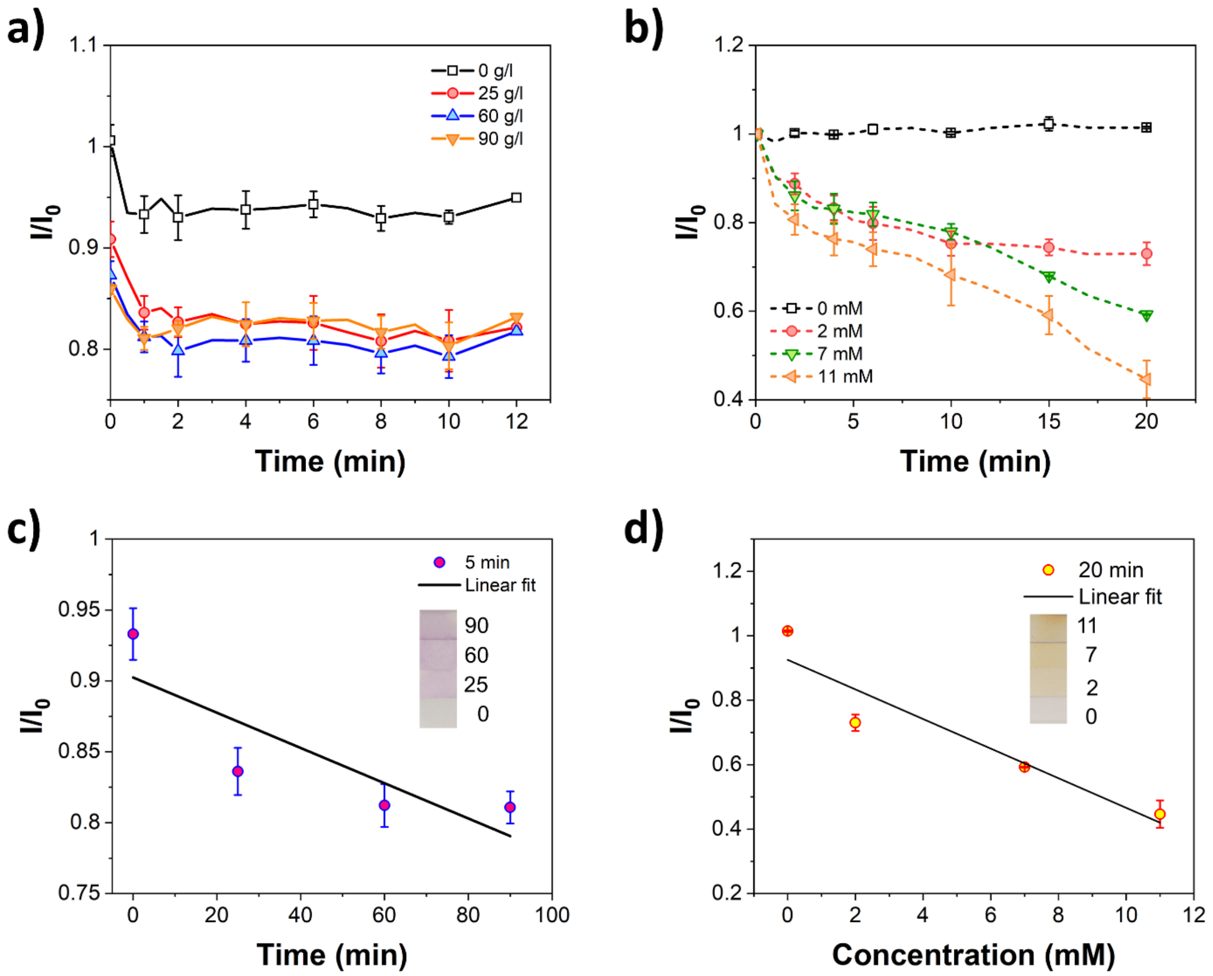
a)
b)
c)
d)
I/I$_0$
Time (min)
Concentration (mM)
0 g/l
25 g/l
60 g/l
90 g/l
0 mM
2 mM
7 mM
11 mM
5 min
20 min
Linear fit

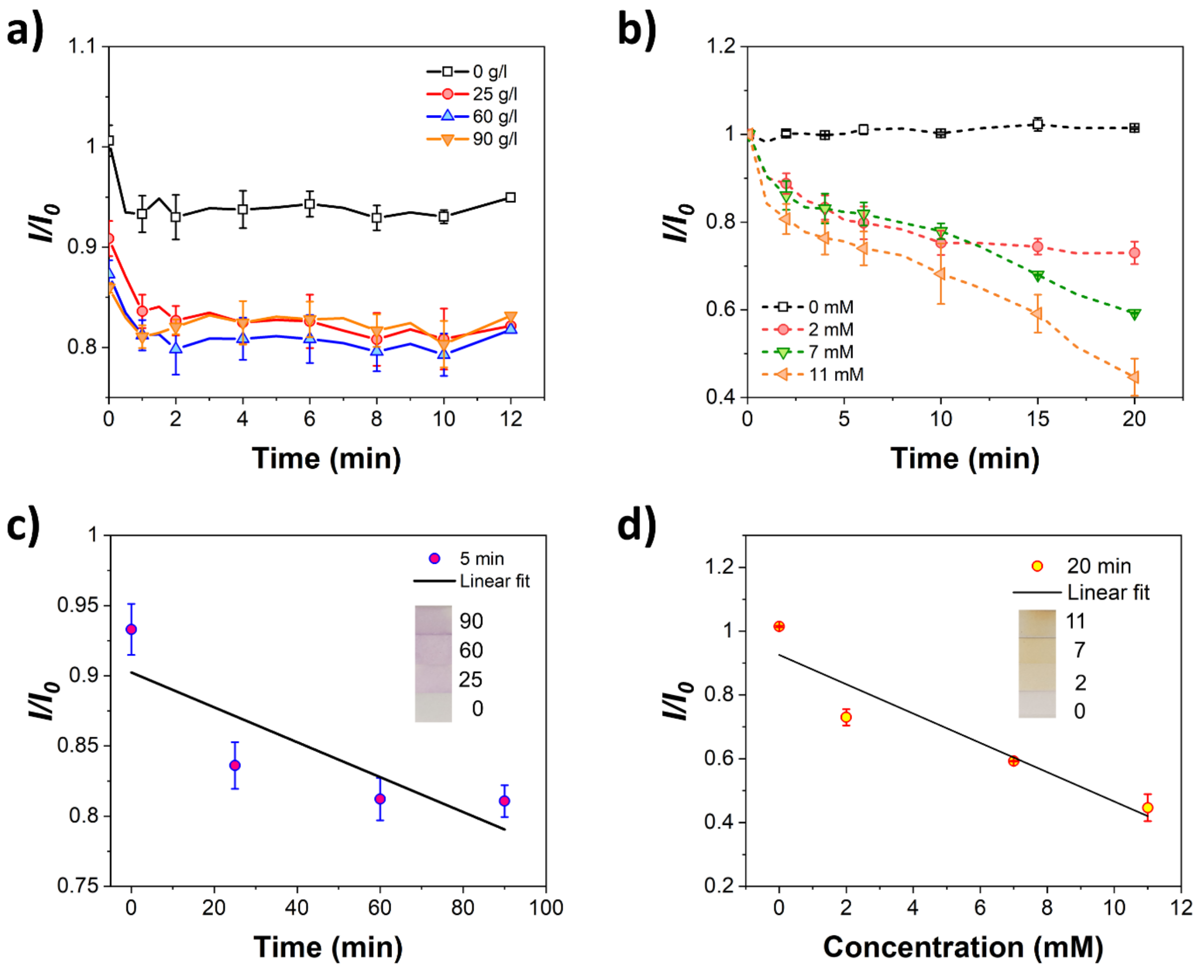

Figure S10. Protein and glucose sensors on filter paper: a) normalized color intensity on the protein sensing area at different BSA concentrations, b) normalized color intensity on the glucose-sensing area at different concentrations, c) calibration curve for the protein assay, inset: color on the sensing areas at different concentrations (unit: g/l), and d) calibration curve for the glucose assay, inset: color on the sensing areas at different concentrations (unit: mM). Curves represent mean ± standard deviation from three parallel samples.

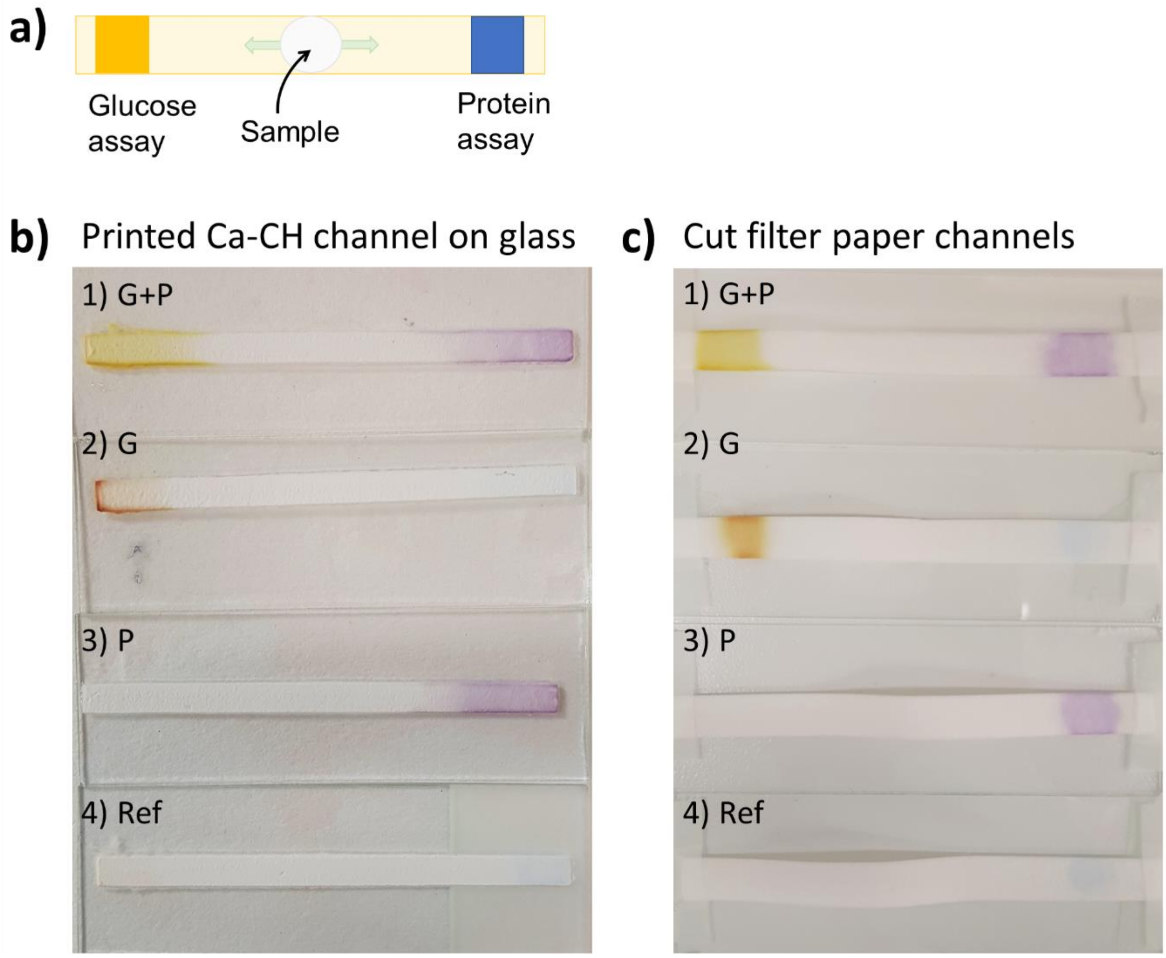

Figure S11. a) Schematic illustration of the multi-sensing assay. b) Multi-sensing assays on hand-printed Ca-CH channels on glass substrates and c) multi-sensing assays on cut filter paper channels. Color responses with different samples: 1) 7 mM glucose and 50 g/l BSA, 2) 7 mM glucose, 3) 50 g/l BSA, and 4) MilliQ-water.

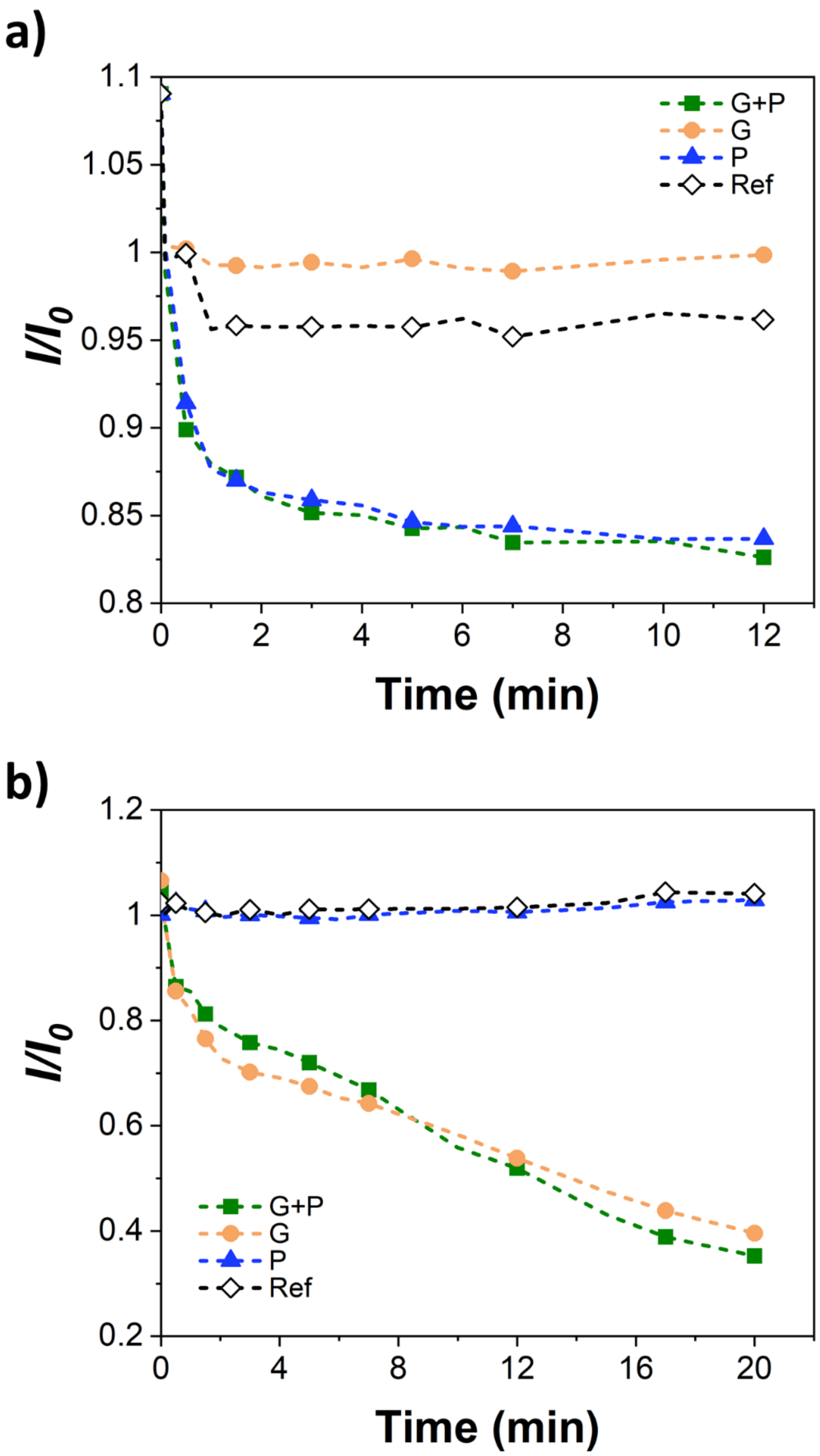

Figure S12. Multi-sensing assays on filter paper: a) normalized color intensities at the protein-sensing areas (right side), and d) normalized color intensities at the glucose-sensing areas (left side). Samples: (G+P) with 11 mM glucose and 25 g/l BSA, (G) with 11 mM glucose, (P) with 25 g/l BSA, and (Ref) with MilliQ-water.
